# X-Ray Computed Tomography: Semiautomated Volumetric Analysis of Late-Stage Lung Tumors as a Basis for Response Assessments

**DOI:** 10.1155/2011/361589

**Published:** 2011-05-24

**Authors:** C. Bendtsen, M. Kietzmann, R. Korn, P. D. Mozley, G. Schmidt, G. Binnig

**Affiliations:** ^1^DECS, AstraZeneca, 50S27 Mereside, Alderley Park, Macclesfield, Cheshire SK10 4TG, UK; ^2^Definiens AG, Trappentreustraße 1, 80339 München, Germany; ^3^Department of Imaging, Merck Research Laboratories, 770 Sumneytown Pike, WP42-305, West Point, PA 19486-0004, USA

## Abstract

*Background*. This study presents a semiautomated approach for volumetric analysis of lung tumors and evaluates the feasibility of using volumes as an alternative to line lengths as a basis for response evaluation criteria in solid tumors (RECIST). The overall goal for the implementation was to accurately, precisely, and efficiently enable the analyses of lesions in the lung under the guidance of an operator. *Methods*. An anthropomorphic phantom with embedded model masses and 71 time points in 10 clinical cases with advanced lung cancer was analyzed using a semi-automated workflow. The implementation was done using the Cognition Network Technology. *Results*. Analysis of the phantom showed an average accuracy of 97%. The analyses of the clinical cases showed both intra- and interreader variabilities of approximately 5% on average with an upper 95% confidence interval of 14% and 19%, respectively. Compared to line lengths, the use of volumes clearly shows enhanced sensitivity with respect to determining response to therapy. *Conclusions*. It is feasible to perform volumetric analysis efficiently with high accuracy and low variability, even in patients with late-stage cancer who have complex lesions.

## 1. Introduction

The standard tool for assessing the response of solid tumors to therapy is X-ray computed tomography (CT). Based on typically axial CT images, the radiologist is faced with the challenge of assessing the tumor burden prior to treatment (baseline) and then following this over the course of therapy. Most clinicians visually compare the scans that were acquired immediately prior to the start of treatment to each new image set obtained during the course of treatment. Their nonquantitative impressions can be sufficient when changes are conspicuous. However, quantification is useful in many patients with cancer, because most solid tumors do not remit or progress rapidly. 

Response Evaluation Criteria in Solid Tumors (RECIST) [1] is currently the standard method for performing quantitative assessments. Currently, RECIST uses the sum of the diameters of the lesions that can be measured before treatment begins as a benchmark. These (target lesions) are then remeasured periodically during a course of treatment. A 30% decrease in the sum of the diameters over baseline is categorized as partial response (PR), while a 20% increase in the sum of the diameters over the previously smallest encountered sum of the diameters for the patient (the nadir) is considered progressive disease (PD). Responses which do not meet criteria for either PR or PD are classified as stable disease (SD). For nonnodal lesions, the longest transaxial diameters should be measured on the slice where the lesion diameter is greatest.

While this system sometimes works well enough, the effectiveness of simple unidimensional measures to quantify the complex changes occurring in lesions over time has been questioned [2, 3]. There is a concern that utilizing only a fraction of the available information in the CT images hampers sensitivity [4, 5]. The lack of sensitivity can degrade the quality of care for individual patients who need to stop futile, but still potentially toxic, treatments as soon as possible. Analogously, more sensitive indicators of response are needed to accelerate the clinical trial process of delivering new treatments to groups of patients with unmet medical needs [6].

A potentially more sensitive and accurate alternative to line lengths as the basis for RECIST would be to measure the actual volume of the target lesions [2, 3, 7, 8]. In fact, this was proposed more than 25 years ago [9] when it was still necessary to manually demarcate the tumor boundary on each axial slice. The primary reason why this approach has not made it into the main stream of clinical practice is that it has been too labor intensive [10]. While some instrument vendors, research groups, and software developers now provide more automated capabilities to do volumetric analysis [11–15], many of the initial reports of precision were disappointing [16, 17]. 

In this paper, the Definiens Cognition Network Technology [18] was used to implement expert knowledge into an image analysis algorithm. The software development framework Definiens Developer XD [19] has been successfully applied to a variety of image analysis tasks based on data from very different kind of sensors ranging from satellites equipped with radar or optical sensors, over electron or optical microscopes to three-dimensional computer tomographs (see, e.g., example applications in [18, 20–24]). The sections that follow, describe the application of the Cognition Network Technology on a semiautomated volumetric analysis of lung tumors. While most other recent publications in the field focus on lung nodules [10, 25, 26] or lymph nodes [27], the focus of this study is to enable the analysis of late-stage pulmonary masses, which tend to be large, irregular in shape and often directly confluent with the borders of other anatomical structures. Accuracy of the semiautomated approach described herein is assessed from the analysis of an anatomical phantom. Intra- and interoperator variability is measured on a set of clinical images from a recent phase III trial of patients with nonsmall cell lung cancer (NSCLC). Finally, the sensitivity of the volumetric analysis is compared to that of RECIST line lengths on the same set of clinical images.

## 2. Methods

### 2.1. Images Analyzed

While there is general agreement that the accuracy of volumetric analysis is inversely proportional to the slice thickness [16], the present goal is to assess the accuracy of the algorithm detailed below in settings that are representative of the images acquired in multicenter clinical trials, where 2.5–5 mm reconstruction intervals are typically what is available. A phantom scan was obtained from scientists at the US Food and Drug Administration (FDA) which was reconstructed at 5 mm intervals without gaps. The phantom contained a variety of elliptical masses embedded in an anthropomorphic model of the human thorax [28]. The model masses analyzed had true volumes between 4.2 and 37 mL and densities between −10 and 100 HU.

10 cases were selected for analysis from an unpublished phase IIb/III multicenter trial with more than 200 patients who suffered from late-stage NSCLC. The images were selected in chronological order of enrollment under the constraint that 5 or more sequential CT scans were available for each case. The target lesions for analysis were required to be predominantly in the lung and to have been previously selected as target lesions by the central radiology laboratory that conducted the formal reading for the trial sponsor. Screenshots of the one slice per case that showed the official longest diameter for each target lesion at baseline were provided to the readers performing the analysis. No other information about the cases was made available to the image analysts.

### 2.2. Cognition Network Technology

The Cognition Network Technology [18] is implemented in the software platforms Definiens Enterprise Image Intelligence and Definiens XD, with the latter being the platform used for the analysis of the present study. Both platforms feature an environment where scripts written in the Cognition Network Language, described in detail below, can be developed and executed. This environment allows the user to load image data, and generate, execute, and edit graphically the analysis script. Results can be visualized, and properties and overall statistics of the resulting three-dimensional image objects (segments) can be exported. The interactive mode allows for rapid script development with a steep learning and progress curve. Medical expert knowledge can be modeled using a class hierarchy (ontology) with fuzzy classifier functions to accommodate uncertainties in the description accuracy and to increase classification robustness in the presence of biological variations. During solution development, the model structure and its parameters are iteratively improved to converge to the required classification and segmentation accuracy, enabling easy integration of new knowledge. The execution environment uses a workspace concept in which the user may process many images off line and— if needed—in parallel on a computer cluster. Definiens XD is a newly developed platform that is specifically designed for multidimensional image data [19]. In particular, it facilitates development and deployment of a wide range of multidimensional image analysis applications within areas such as medical imaging and preclinical small animal imaging.

### 2.3. Cognition Network Language

The Cognition Network Language (CNL) [18] is an object-based procedural computer language which is specifically designed to enable automated and semiautomated implementation of complex, context-dependent image analysis tasks. It consists of four basic data structures: *processes*, *domains*, *image objects,* and *image object classes*. The language was designed to provide an easy to learn but powerful approach to specify a complex image analysis task through the combination of less complex tasks. Each language element representing the dynamic of the analysis is called a *process*. There are *processes* to manage *image objects*, object features, *classes*, and variables, file input/output, image filters, segmentation, object linkage and classification operations, and control structures such as conditional execution commands and loops. The *processes* are organized in a process tree hierarchy.

The *process* execution engine recursively executes a root process and then subsequently all of its child *processes* in a depth-first order. By selecting and parameterizing the *processes,* the particular processing algorithms are specified for a given programming step, whereas through the definition of a *domain* the system is guided to the data structure that is going to be processed. The *processes* define what and the *domains* specify where processing takes place. The most important *domains* are *pixel level domains* for filtering and initial segmentation operations, the *image object domain* for *processes* which operate on *image objects* (segments) with specific classifications and properties, and the *image object relation domain* which allows the navigation in the *image object* network. Navigation is particularly useful to process the neighbors or subobjects of a given *image object* in the current *process* with the algorithms in its *subprocesses*. 

An *image object* represents a group of voxels or a group of *image objects*. An *image object* comprises methods to calculate its properties such as shape, position, mean spectral values, or texture. Since *image objects* may be linked to other *image objects* using specific *processes*, relational properties such as relative surface contact area or relative brightness can be easily computed and used in the processing. *Image objects* are either generated by basic segmentation (e.g., multiresolution segmentation [22]) or by grouping existing *image objects* on a higher *image object* level. The Cognition Network Language provides operations to resegment and to reclassify *image objects* with specific, potentially context-dependent properties. Each *image object* may be classified according to the fuzzy membership functions defined in the *image object classes*.

*Image object classes* describe the kind of objects to be searched for in a given image. The *classes* may be grouped in a class hierarchy to enable the execution of *process* operations on groups of *classes*. Each *class* carries a name, a visualization color, and optionally a logical expression of fuzzy membership functions. The membership functions are piecewise linear functions which describe the contribution of each specified *image object* property (e.g., area, brightness, distance to another *image object* with a given classification) to the overall class membership. *Image object classes* without membership function may be used as labels which can be assigned to an *image object* using a class assignment *process*.

### 2.4. Semiautomated Analysis Workflow

**Figure 1:**
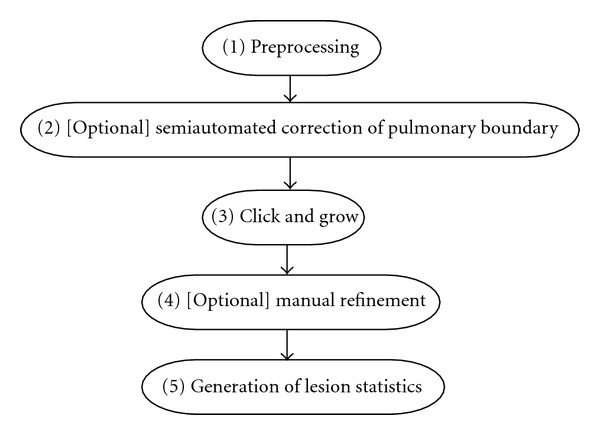
Flowchart depicting the semiautomatic analysis workflow.

The overall goal for the implementation was to accurately, precisely and efficiently enable the analyses of lesions in the lung under the guidance of an operator. A standard analysis workflow was established using the Cognition Network Technology. The workflow contained the following elements and is summarized in Figure 1.

**Figure 2:**
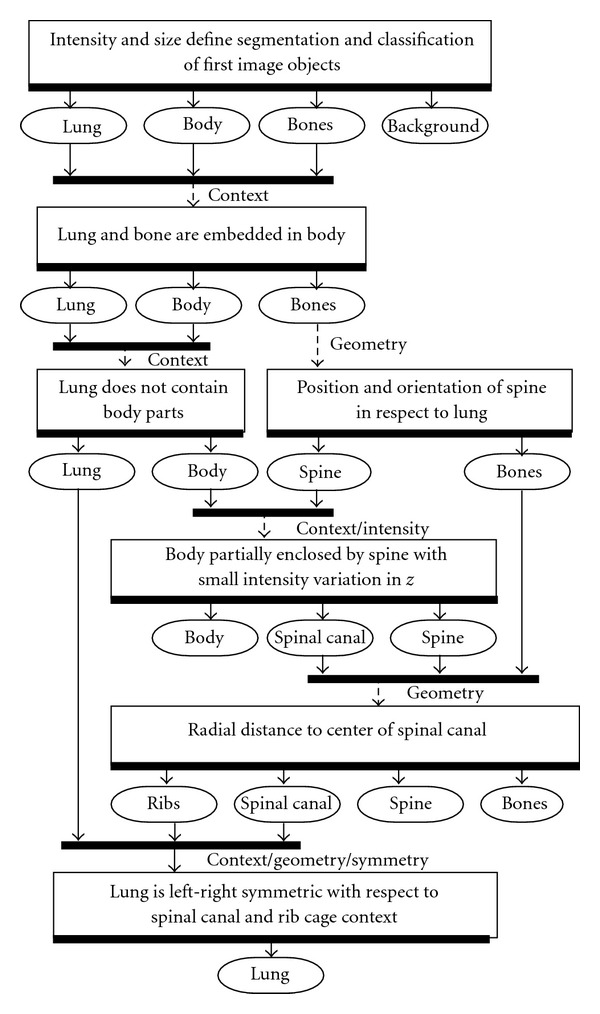
Flowchart showing image object manipulation during preprocessing. Rectangles show the used domain knowledge, whereas rectangles with rounded corners show segmented and classified image objects.

**Figure 3:**
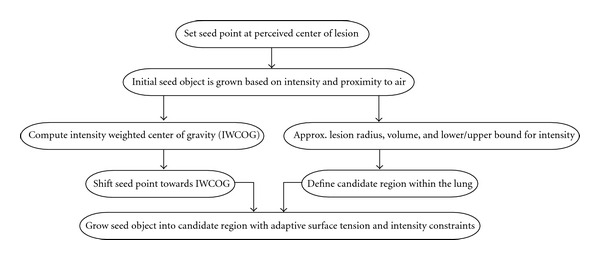
Flowchart of the seed-based segmentation of the lesions.

1. A preprocessing step (Figure 2). This was designed to perform a segmentation of the lung as well as other offline tasks, such as filtering, to improve the interactive performance of the analysis.
2. An optional step with semiautomated correction of the segmented lung. Since lesions are commonly found to be attached to the pleural surface, it was critical to enable efficient correction of the lung boundary in cases where the boundary between juxtapleural target lesions and the pleura had not been correctly determined during the automated preprocessing step.
3. A “Click & Grow” step with seed-based segmentation of the lesions (Figure 3).
4. An optional manual refinement step of the semiautomated lesion segmentation to ensure medical expert agreement with any results that could influence patient management.
5. A reporting step generating volumes and statistics about other features, such as average density.

The details of each of the above steps are given below.

**(1) Preprocessing**

The preprocessing performed automated organ segmentation with the main goal of segmenting the aerated lung with correct identification of the pleural wall in order to easily facilitate the semiautomated segmentation of juxtapleural lesions.

Context objects such as lung, spine, and ribs do not require the same precision as objects used for quantification. Therefore, in order to improve offline performance, context objects were segmented and classified automatically on a downscaled image without user interaction.

**Figure 4:**
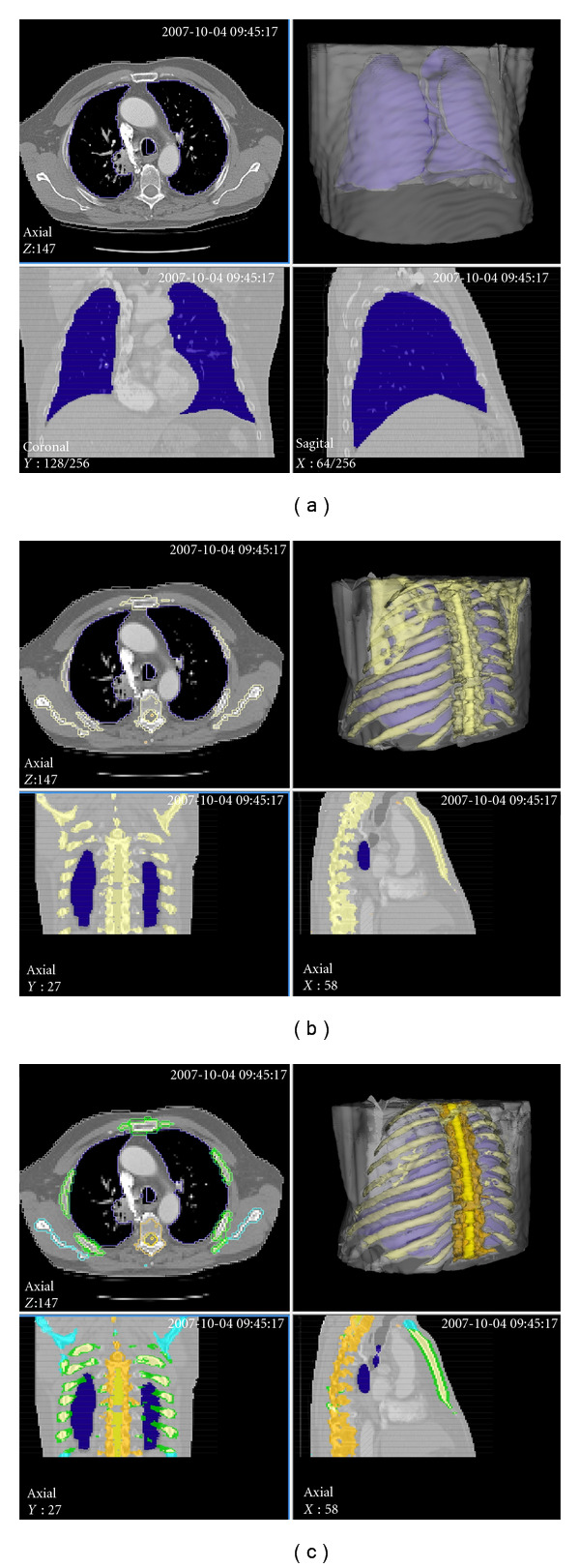
Three stages of preprocessing to generate context for subsequent lung tumor detection. (a) Segmented lung (blue) and body (gray). (b) Segmented bones (yellow). (c) Segmented ribs (light yellow with green coating), spine (dark orange), and spinal canal (light orange).

In a first step body, background, lung, and bone were identified based on intensity and object size (Figures 4(a) and 4(b)). Moreover, context constraints were implemented which ensures that lung and bone objects are embedded in body and lung does not contain body parts. In a second step, parts of bone were subsegmented into spine according to geometry constraints imposed by position and orientation of the detected lung. In a third step, those parts of body which are partially enclosed by spine and show small intensity variation along the *z*-axis were segmented as spinal canal. The processing imposed the existence of a single, deformable pole like spinal canal without branches. Geometric information about the detected spinal canal such as radial distance to its center in each slice was used to refine the spine and to subsegment bone to ribs in a fourth step (Figure 4(c)). Consecutively, the segmentation of the lung was improved by leveraging left-right symmetry and using the geometry of the spinal canal and the rib cage as context. Finally, the image was upscaled to its original size and the lung segmentation was refined, removing aliasing effects at its border. The flowchart in Figure 2 outlines the entire process.

**(2) Semiautomated Correction of Pulmonary Boundary**

In order to perform the seed-based segmentation of a target lesion, the latter has to be completely within a lung *image object*. In cases where a medical expert concluded that the automated preprocessing described above failed to accurately identify the border between a target lesion and the pleural wall, it was necessary to enable correction of the automated lung segmentation. To this end, the image analysts identified the part of the lung that needed modification and placed a seed point manually where the segmentation should be corrected. A seed point outside the lung defined a lung extension, whereas a seed point inside the lung defined a reduction.

In case of a lung extension, a two-dimensional, axially oriented convex hull is constructed using lung voxels close to the seed point and the seed point itself. All body voxels within that convex hull were added to the existing lung object. Finally, corrections to the lung segmentation on this slice were extended to slices above and below by region growing in the *z*-direction into body voxels with similar intensity compared to the convex hull body voxels.

In case of a lung reduction, two different distance measures were used. First, all lung voxels within the same axial slice as the seed point were identified by converting the three-dimensional lung object into a set of axially oriented two-dimensional slice objects and using the neighbor relation to the seed point object for identification. Next, the distance of each of these voxels to the center of gravity as well as the border of the 3D lung object was calculated. Utilizing this information, the seed point was grown into voxels with a smaller distance to the lung border and a greater distance to the center of gravity. Subsequently, these voxels were removed from the original lung object. Finally, corrections to the lung segmentation on this slice were extended to slices above and below using region growing in *z*-direction.

**(3) Click and Grow**

In order to segment a target lesion the image analysts identified the lesion, within the segmented lung and placed a seed point in its interior—typically at the perceived center of the lesion. Starting from the seed point, an initial seed object was automatically segmented using region growing based on similar intensity and proximity to areas with low intensity (“air”). From this small sphere of voxels with similar density the intensity weighted center of gravity (IWCOG) was calculated. To decrease inter- and intrareader variability, the seed point was shifted closer to the IWCOG. Additionally, an approximation of the lesion radius and volume, and a histogram-based lower and upper bound for the intensity were extracted. These parameters were used to define an octahedron-shaped candidate region within the lung. The seed object was then grown into the candidate region with adaptive surface tension and intensity constraints. This Definiens proprietary region growing process approximates the object's surface tension *T* using an *N*^3^ voxels sized kernel locally by calculating the ratio of the object volume inside a kernel (*V*_*i*_) to the total kernel volume (*V*_*K*_), *T* = *V*_*i*_/*V*_*K*_. With this approach, a high relative kernel volume of the objects surface voxels corresponds to a high surface tension. The strength of the surface tension was mainly controlled by the volume of the growing object in order to impose a smoother surface for larger objects. The flowchart in Figure 3 summarizes the above process.

The intensity constraints restrict the growing into candidate regions defined by (1) a precomputed intensity range of the Gaussian-smoothed CT image, where the intensity range was estimated from the intensity statistics of the seed region and (2) a bound on the distance to the seed region which was calculated using a distance map. The distance map was calculated solely for the candidate region within the CNL local processing framework and provides the minimal distance for each voxel to the seed region as an intensity value. Using the distance map ensures an approximate convexity of the growing seed object when growing into regions with similar intensities.

If the growing process did not sufficiently capture the target lesion, the operator could place additional seed points within the lesion and repeat the growing process outlined above. Upon completion of the segmentation, the individual image objects were merged to form a single image object representing the segmented target lesion.

**(4) Manual Refinement and Generation of Lesion Statistics**

Upon completing a seed-based lesion segmentation as described above, medical experts were provided an opportunity to certify the results by scrolling up and down the stacks of axial images to verify that the segmentation followed the anatomical compartment boundaries properly. Rules for conceptualizing and visualizing the borders were not formally established in chronological order, but included review of the lateral pleural surfaces, the mediastinal surfaces, and the diagphragmatic surfaces. Special attention was required for masses that invaded the mediastinum to prevent the segmentation from either misclassifying neoplastic tissue as normal soft tissue or keeping the algorithm from “running away”, that is, from classifying normal soft tissue with nearly the same attenuation as the mass as part of the neoplastic mass.

To facilitate medical expert certification of the seed-based growing algorithm, tools were constructed of two types. One type allowed the operators to limit the boundaries beyond which the region could grow during the “Click and Grow” step by manually placing “blocker” points. The combination of distance transformation and location of the blocker points was used to determine spherical caps. The size of the caps were defined by a parameter called the blocker size and the caps functioned as a barrier during the growing process. Another type enabled manual editing through the standard Developer XD Platform functionality. This allowed for manual editing of the contour of each segmented lesion on each axial slice by cutting, merging, and reclassifying objects and thus gave the image analysts full ability to perform any desired modifications of the segmented lesion.

Image analysts were empowered to override as much or as little of the semiautomatically grown regions as their expertise suggested was indicated.

Once the segmentation of all target lesions was deemed sufficiently accurate by the image analysts, statistics for each lesion, such as volume, center of gravity, and average density, all readily available as object features within the Cognition Network Language, were reported. The object feature used for reporting lesion volume simply summed the volume of all voxels constituting the object representing a segmented lesion. Additionally RECIST diameters were automatically computed by determining the maximum distance between any two lesion voxels within an axial slice for each of the axial slices and reporting the highest value as the RECIST longest diameter.

### 2.5. Statistical Analyses

Operator accuracy was calculated as the ratio of the segmented image object volume to the true volume of the object. True volume was given by scientists at the FDA, who used physical methods to certify that the volumes of the objects were accurately reported by the manufacturer of the thoracic phantom.

Intra- and interoperator variabilities were calculated as the relative difference to the mean; agreement was assessed using concordance correlation coefficients [29]. For the clinical cases, true volume was not known. Operator variability was further characterized using Bland-Altman plots [30]. Differences between RECIST and volumetric measurements were assessed using the log rank test. All statistical analyses were conducted in R [31].

## 3. Results

Image analysis of the anatomical phantom showed that Reader 1 obtained an average accuracy of 88% (95% CI 77% to 101%). The results for Reader 2 were almost identical, leading to an overall average accuracy of 88% (95% CI 77% to 101%). These results were obtained without allowing manual refinement of the automatically grown regions. When allowing this, the average accuracy improved to 95% (95% CI 90%–102%) for Reader 1 and 98% (95% CI 94% to 102%) for Reader 2 for an average accuracy of 97% (standard deviation 4.8%, 95% CI 90%–103%). Interreader agreement was excellent with an average postmanual editing interreader variability of 4.0% (upper 95% CI of 9.5%) and a concordance correlation coefficient of 0.9996.

**Figure 5:**
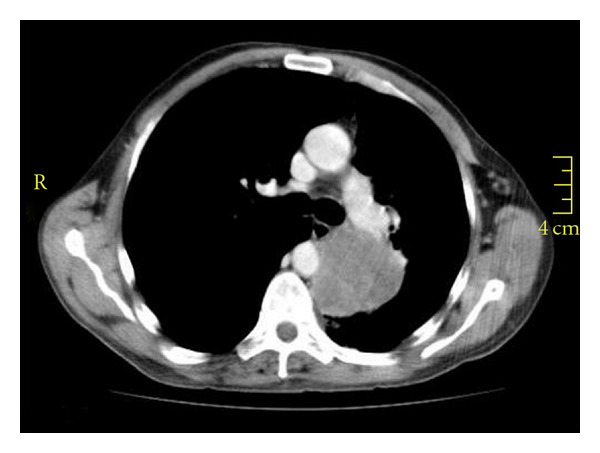
Representative example of a clinical case showing axial slice of baseline scan with target lesion.

The clinical cases were found to be heterogenous. Slice distances, or reconstruction intervals, varied between 1 and 5 mm, with a mean of 4.15 mm, and a slice thickness generally equal to the slice distance (range 1 to 7 mm, mean 4.97 mm). A total of 71 scans were provided for analysis, with a total of 100 analyzed target lesions. Of these, 75 were attached to the pleural surface and 72 showed clear spiculation. Only 12 analyzed lesions were without visually obvious spiculation and without bordering the pleural surface, and 10 of these 12 showed visual connections to large pulmonary blood vessels. A representative example of the lesions analyzed is shown in Figure 5.

The tumors were analyzed independently by two readers following the workflow described in the Methods section. The findings showed that the masses had an average volume of 34 mL (range from 0.04 mL to 381 mL). Target lesion volumes were summed for each scan to produce a sum of the volumes (SV) similar to the RECIST sum of the diameters. While no explicit guidelines on time usage was given to the readers, Reader 1 spent an average of 8.4 minutes per time-point assessment while Reader 2 spent an average of 14 minutes. Reader 1 additionally redid the analyses in order to enable assessment of intra-reader variability. All analyses were conducted using a window setting of −500 HU to +500 HU.

The mean intra-reader SV variability was 5.0% (upper 95% CI of 14%), and the mean interreader SV variability was 5.3% (upper 95% CI of 19%). Both intra- and interreader agreement was excellent with a concordance correlation coefficient for both of 0.993.

**Figure 6:**
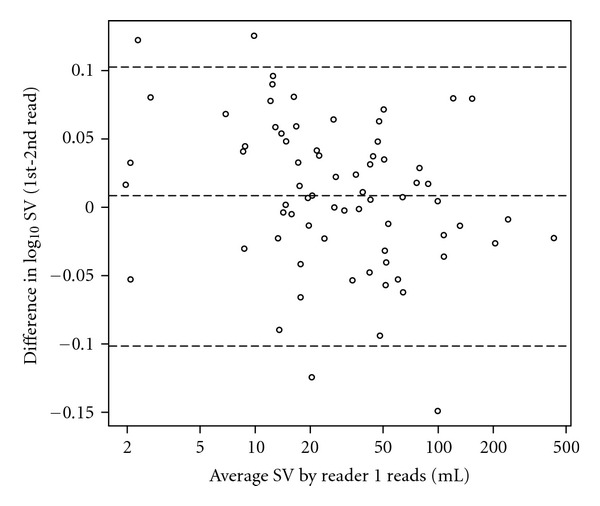
Bland Altman plot showing intra-reader variability as the average volume of the Reader 1 reads versus the difference between the log base 10 transformed volumes of the reads. The lines show the median and the 95% confidence interval.

**Figure 7:**
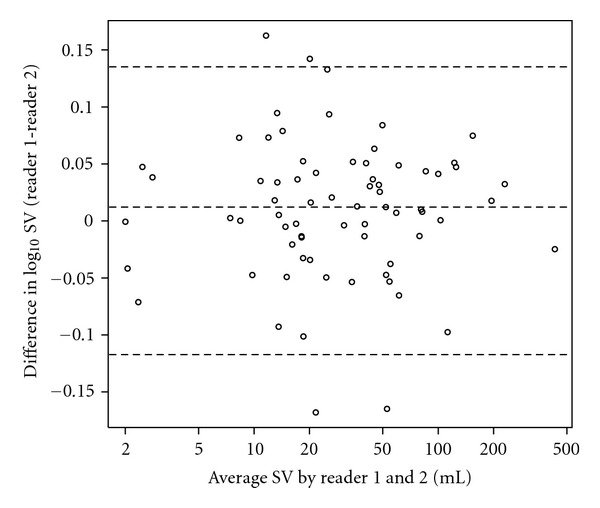
Bland Altman plot showing interreader variability as the average volume of Reader 1 and Reader 2 versus the difference between the log base 10 transformed volumes of the readers. The lines show the median and the 95% confidence interval.

Bland-Altman plots of the intra- and interreader volumes are shown on Figure 6 and Figure 7, respectively. Very little bias is observed for both intra- and interreaders (2.0% and 2.8%, resp.) and the 95% CI of the ratio between the volumes minus one for the two Reader 1 reads is from −21% to 27%, whereas for the volumes of Reader 1 and Reader 2 it is from −24% to 37%.

From the automatically generated RECIST line lengths based on the segmented volumes, one observes high agreement between Readers 1 and 2 as measured through a concordance correlation coefficient of 0.96. The mean interreader variability was similar to what was observed for the volumetric analysis, irrespectively of the fact that the dynamic range of the line lengths was much smaller than of the volumes.

**Figure 8:**
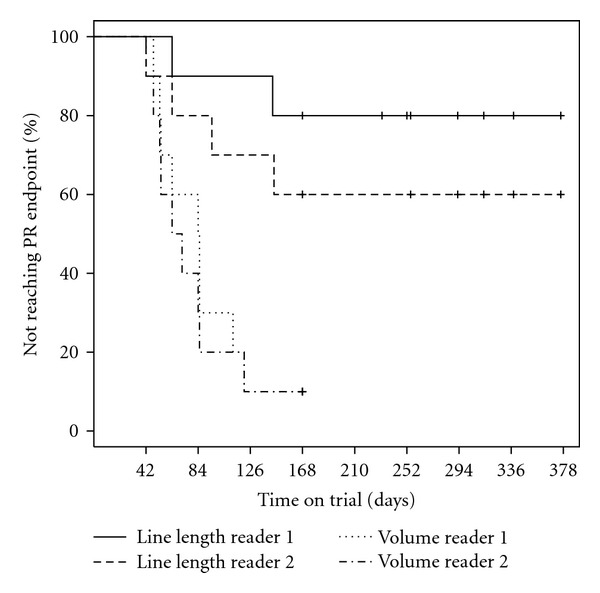
Kaplan-Meier curves of time to partial response (PR) for Readers 1 and 2 of the 10 clinical cases for both sum of the volumes and sum of the longest diameters. Censoring events are indicated by “+”.

**Figure 9:**
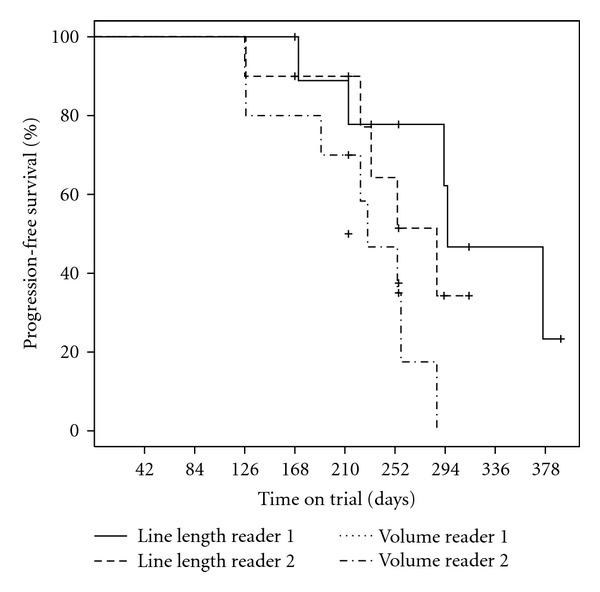
Kaplan-Meier curves of progression-free survival (time to PD) for Readers 1 and 2 of the 10 clinical cases for both sum of the volumes and sum of the longest diameters. Censoring events are indicated by “+”.

In order to determine if a volumetric analysis is more sensitive than the classical unidimensional line lengths for the clinical cases analyzed, both time to partial response as well as progression-free survival was assessed for the two readers independently. Given the facts that (1) the accuracy with the volumetric analysis of the phantom was high, (2) concordance between readers was better for volumetric analysis than RECIST line lengths, and (3) the interreader variabilities were comparable, the volumetric analyses conservatively used identical −30% and +20% thresholds to those of line lengths by RECIST for determining PR and PD. Figures 8 and 9 show the Kaplan-Meier curves for time to PR and time to PD, respectively. Using the log-rank test to test the null hypothesis of no difference in time to PR by diameters versus volumetric analysis, the null hypothesis is rejected for both readers (*P* = .002 and *P* = .016, resp., two-sided *P*-values, exact log rank test). For progression-free survival, the null hypothesis is similarly rejected for Reader 1 (*P* = .02) but not for Reader 2 (*P* = .14).

## 4. Discussion

The present study set out to test a semiautomated workflow for volumetric quantification of neoplastic masses in the lungs which are typical of late-stage NSCLC. The workflow relies on radiological expertise to initiate and certify the segmentation process, but aims at being time efficient, accurate, and precise.

The results show that volumetric analysis can be performed with high accuracy, especially when allowing manual refinement of the semiautomated segmentation. Assessments of accuracy were based on relatively low resolution, thick slice CT scans, which are still typical of what is available in global clinical trial settings. They apply to lesions which are sufficiently large to be visible on several slices. This setting is somewhat different than the analysis of lung nodules in diagnostic settings, where thin slice CT has been used to compute volume doubling time [32]. The observed high average accuracy of 97% and low standard deviation of 4.8% are better than previously reported for small nodules [33] and consistent with the error expected solely due to acquisition [34].

The results for these 71 time-points with late-stage NSCLC show that it is feasible to perform time efficient volumetric quantification of complex masses under the appropriate guidance of an experienced operator. Low intra- and inter-operator variabilities were obtained with a mean of approximately 5%. While not specifically assessed, this is considered due to the semiautomated approach which tends to let the operator focus on the major features of the segmentation rather than exactly outlining the border of a lesion on each and every slice. The Bland-Altman plots indicate no specific factor of variability due to lesion size which, in contrast to what is reported in for example, [10, 16, 35], is possibly due to the facts that (1) none of the patients analyzed at any given time had sum of volumes which were below the milliliter range and (2) some of the masses had very complex geometries and, at least in this setting, complexity was not directly proportional to size. Post hoc inspection of the segmented boundaries suggest that the outliers in variability are mainly due to judgment calls of the operator; as for example, cases with pleural edema bordering the target lesions did not enable a clear separation of lesion from edema based on attenuation alone and thus required operator judgment on where to draw the border between the two. In reality, this kind of variability is of little concern with respect to following disease progression as long as the same judgment is applied throughout the longitudinal analysis of any given patient, and as concluded in [3] it is therefore preferable to have the same operator perform the entire analysis for any given patient.

RECIST establishes categorical responses based on predefined thresholds for the relative change in line length to baseline or nadir. The question when applying an equivalent approach for volumetric analysis is thus what thresholds should be used. If one assumes that a tumor is shrinking or growing symmetrically in all three dimensions, then the −30% or +20% thresholds for line lengths correspond to an approximately symmetrical volumetric change of −66% and +73%, respectively. Adopting this as a threshold for volumetric analysis seems overly conservative, especially since lesions are generally not expected to contract or expand uniformly. The question at hand is thus which thresholds can be applied without risking misclassification due to operator variability. From the phantom accuracy study, one seemingly could safely adopt a threshold of ±10%; however, considering the interoperator variability obtained on the 10 clinical cases with an upper 95% confidence interval on the variability of 19%, a threshold of ±20% seems more reasonable. As this, however, does not take into account the fact the variability is in part due to operator judgment, as discussed above, one may in fact be able to use a lower threshold provided that the analysis protocol stipulates that consistent judgment on uncertain boundaries should be maintained throughout the longitudinal analysis of any given patient. Conservatively, this study adopted identical thresholds for volumes and longest diameters as also done in [13]. In addition to what is mentioned above, this is further supported as conservative thresholds by the fact that, consistent with [36], better agreement was observed between volumes than the unidimensional line lengths and that the interreader variability for the volumetric analysis was approximately identical to that of longest diameters, even though the range of changes observed for the volumes were much larger than for the unidimensional line lengths.

The comparison of volumetric analysis to line lengths as the basis for RECIST in the clinical cases analyzed herein reveals that volume is significantly more sensitive with respect to detecting patient response to therapy. In the survival analysis of progression-free survival, a significant difference to longest diameters was established for one reader but not the other, suggesting that volume is more sensitive but that 10 clinical cases are insufficient to show this consistently. The Kaplan-Meier plots additionally show that the volumetric endpoints are less variable between readers than the longest diameters, further confirming that the thresholds used are conservative for those parts of RECIST that depend on longitudinal assessments of target lesions.

In summary, the results show that it is feasible to perform volumetric analysis efficiently with high accuracy and low variability in the context of advanced NSCLC with complex lesions. While a semiautomated approach is critical for efficient analysis and the reduction of operator variability, it is vital for patient safety to make manual refinement and segmentation possible in order to handle complex cases where judgments about where to draw the lesion boundary are deemed medically indicated. 

While additional studies as well as a comparison between the approach presented herein and other approaches for performing, volumetric analysis still remains to be done in order to determine guidelines and extent of standardization necessary for volumetric tumor quantification, the findings suggest that the time is ready for the use of volumetric analysis in the context of patients with solid tumors of the chest.
